# Maintaining Postgraduate Healthcare Education During COVID-19: Does a Virtual Format Allow Effective Engagement and Personal Development?

**DOI:** 10.7759/cureus.19066

**Published:** 2021-10-26

**Authors:** Sharan Sambhwani, Awf Alshahwani, Loukas Andritsos, Nomaan Sheikh

**Affiliations:** 1 Trauma and Orthopaedics, Kettering General Hospital, Kettering, GBR; 2 Trauma and Orthopaedics, Leicester Royal Infirmary, Leicester, GBR; 3 Trauma and Orthopaedics, Northampton General Hospital, Northampton, GBR

**Keywords:** surgical education, team morale, pandemic, journal club, covid-19, coronavirus, virtual education

## Abstract

Introduction

The COVID-19 pandemic has brought unprecedented challenges in health care, leading to a dramatic change in service provision and impacting surgical training. The availability of a virtual meeting platform allowed our team to develop a new educational programme aiming to maintain an ethos of education safely, focusing on providing an opportunity to develop non-technical skills and maintain reflective practice.

Materials and Methods

Microsoft Teams was used to conduct two streams of weekly education: a journal club focussing on developing critical evaluation skills, and case-based in-depth discussion forum to develop presentation skills and evidence-based management. A questionnaire after 10 weeks was used to evaluate the effectiveness and engagement of the two streams.

Results

Fifty-three responses were received. Seventy-two percent felt that their engagement in teaching was increased on a virtual platform. There was 88% satisfaction with the platform. Reflective practice increased and 40% of respondents felt their non-technical skills improved. Sixty-eight percent stated that they would like to continue the virtual format going forward and 88% would recommend this to their peers. Ninety-two percent felt that the platform played a pivotal role in helping maintain team morale during this period.

Conclusion

Our experience of using a virtual tool to maintain education within our department is very positive. There has been good engagement with positive reflection and learning at a time of great change in the NHS. Both trainees and non-trainees have benefited. With ongoing COVID still influencing clinical practice, we recommend utilising virtual platforms to maintain education in surgical departments.

## Introduction

Since the outbreak of COVID-19 and its classification as a pandemic, rapid responses have led to dramatic changes in healthcare provision with potential impact on surgical training. With restructuring of acute services, staff redeployment and cancellation of elective services, it has inevitably led to 'restricted training' in secondary care. Although understandable, pure service provision if sustained will have a definite impact on trainees and their progression in their chosen career.

The health service has a responsibility to maintain a safe work environment for all staff and social distancing measures have been adopted, leading to a halt in the use of traditional teaching formats, either in theatres or in lecture-based settings. Centralised regional programmes for surgical trainees were paused within our region and overall this has led to a decline in academic activities within our Trust.

A fixed-location-based face-to-face session is currently not possible with the change in staff rotas and redeployment across all levels in the medical workforce [1]. The impact of e-learning using virtual platforms has been looked into over the last decade, with encouragement given in developing infrastructure for this purpose [2].

Regional surgical networks utilised virtual meetings in discussing complex cases amongst specialists to standardise care and achieve optimal outcomes for the patient as well as share experience amongst surgeons [3,4]. Virtual learning was started worldwide from the early days of the outbreak in China and as the spread was formally defined as a global pandemic. This was applied from the level of primary schools, through to university degrees. Webinars and online discussions were carried out on a more widespread basis and were accepted by the attendants. Variable platforms and software have been utilised for this purpose [5-7].

Microsoft Teams was introduced into our Trust to allow secure communication in March 2020. To try and maintain some education during a period of rapid deployment and work pattern changes, and to maintain a clear communication stream within the department, we developed a tailor-made educational programme during this period. To facilitate this, we utilised the platform of Microsoft Teams. In this study, we aimed to evaluate the impact of a virtual program on our staff, focusing on the level of engagement, its effectiveness and development of non-technical skills at a time when practical skills were limited.

## Materials and methods

The study performed was prospective, data were collected and analysed by two assessors. The data were analysed with MS Excel, which was also used for the figures' creation. 

We carried out an anonymous survey after 10 virtual educational sessions to evaluate the service, with questions based on Likert scales. In total, 53 out of 63 staff members, who attended the teachings, participated in the survey including Trauma Nurse Practitioners, Foundation year 1 and 2 doctors, Core surgical trainees, Trust Grade Senior House Officers, Training and Non-Training Specialty Registrars, Associate Specialists and Consultants. Our primary outcomes were the satisfaction and engagement of the participants with this virtual teaching compared to the traditional face-to-face sessions. Secondary outcomes we assessed were the ease of access with the technology, the development of non-technical skills including presentation, communication and research skills, and the overall perception of the department in maintaining virtual education in a post-pandemic period.

Ten sessions were run twice a week over a three-month period. The platform used was Microsoft Teams, which is our Trust's online application for all virtual events (teaching, meetings, e-learning). All sessions were moderated by consultants with a timetabled involvement of junior doctors leading the session to allow everyone an opportunity to present. Two independent types of sessions were run during this period addressing two aspects of virtual education: research methodology via Journal Club and clinical reflective practice via Case-Based Discussions along with current concepts. 

The format of all teaching sessions included a 10-minute presentation on a selected subject, and at the end of each session a feedback form was distributed for the assessment of the individual. The content of the presentation and the quality of the slides were assessed from 1 to 5. Also, free text feedback was given about the presentation skills of the individual. 

A mixture of elective and trauma-based topics were used to address the breadth of the Trauma and Orthopaedic Curriculums dictated by the Intercollegiate Surgical Curriculum Programme [8]. Topics were ranging from general orthopaedics to more specialised modules and were allocated based on the moderating consultant interest. An initial emphasis was placed exploring evidence pertaining to trauma, relevant to the change in practice during the pandemic period. Lessons learned were discussed and applied to incorporate how modern practice could be made safer with evidence-based medicine [9]. 

The topics were selected one week in advance allowing preparation time and any supplementary learning links were shared via Microsoft Teams to encourage further self-directed learning. The same questionnaire was used to collect data for both streams of virtual teaching.

## Results

**Figure 1:**
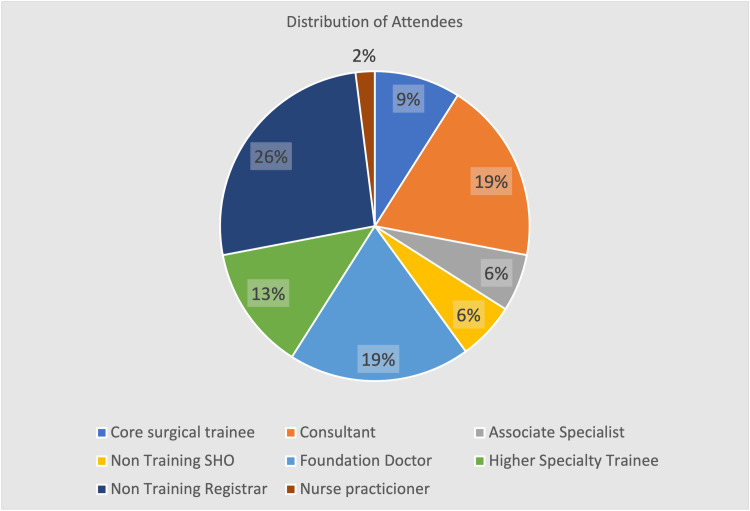
Distribution of attendees

There was a 84% complete response rate, with a total of 53 responses out of 63 survey invites. There was a representative attendance from all levels within the department, including training and non-training clinical participants. Of all attendees, 73% comprised the more junior members of the team, below associate specialist level, as seen in Figure 1. There were also participants with a keen interest in orthopaedics who did not work within the main orthopaedic department at the time but attended sessions regularly.

**Figure 2:**
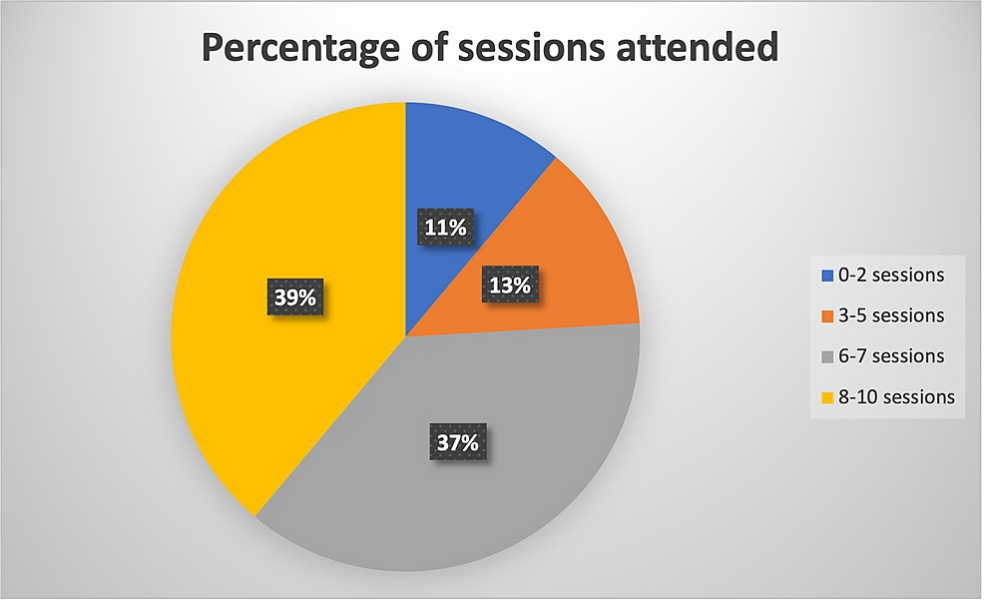
Percentage of sessions attended

**Figure 3:**
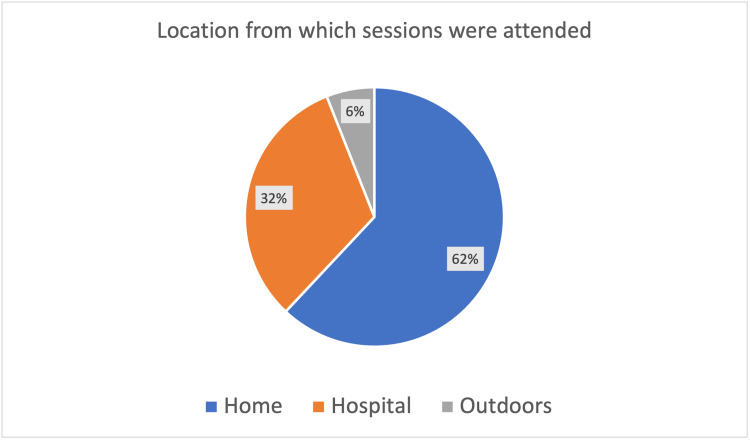
Location from which sessions were attended

**Figure 4:**
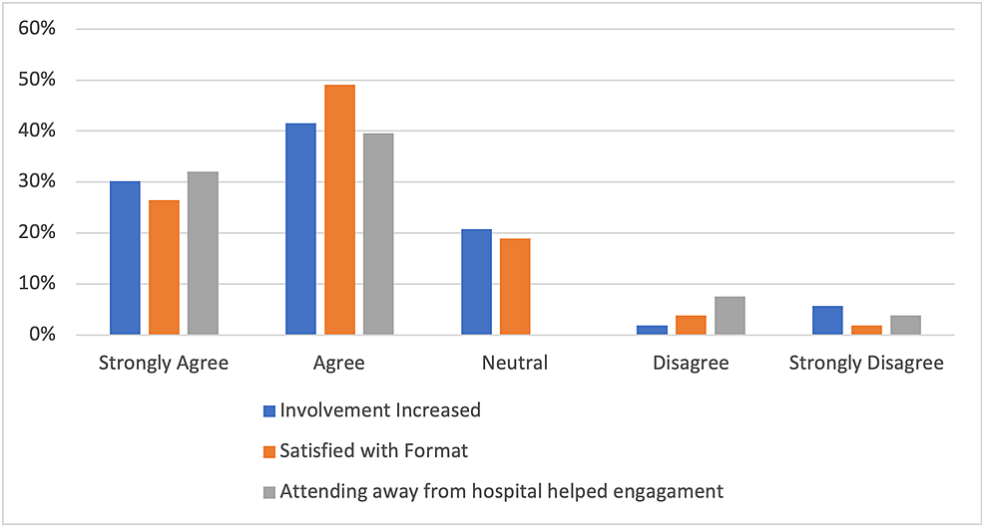
Engagement with sessions

Participants engaged regularly with 78% (Figure 2) attending more than 50% of the time, despite the variations in their new working shift patterns. The majority of attendees (72%) felt that their attendance had increased following the switch to a virtual platform and 75% were satisfied with the format of the sessions. Attendees joined sessions primarily (68%) from outside of the working environment (Figure 3) and agreed (72%) that by doing so they increased their engagement with virtual educational activities (Figure 4). 

**Figure 5:**
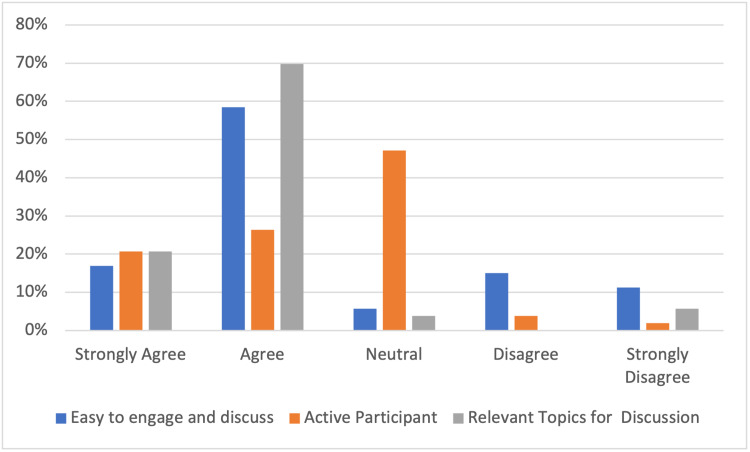
Engagement with sessions

Most participants felt that it was easy to access (81%) and engage (81%) with educational activities and there was a good proportion of regular active participation (47%) during sessions (Figure 4). Almost all (91%) participants felt that topics discussed were tailored and relevant to their educational curriculum (Figure 5). 

**Figure 6:**
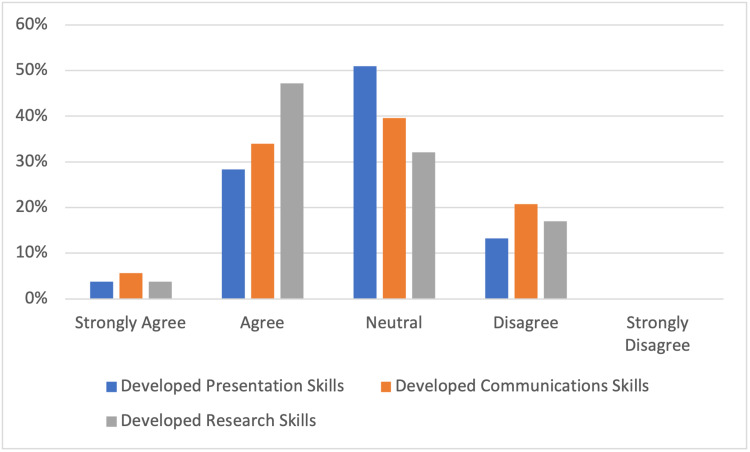
Review of non-technical skills

There was some improvement in non-technical skills in participants (Figure 6) with 32% feeling that their presentation technique improved. Communication (40%) and critical evaluation of research (51%) also improved throughout the three-month period as demonstrated in Figure 6. 

**Figure 7:**
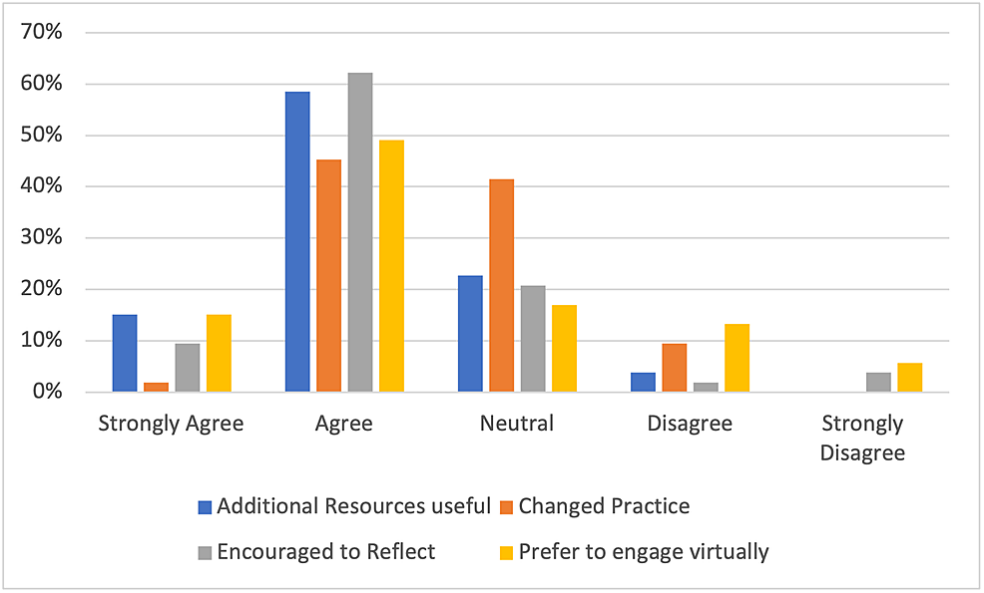
Effect on reflective clinical practice

Participants also engaged in regular (60%) self-directed learning (Figure 7) with many (73%) feeling that the additional resources provided better targeted their learning and development. As a result of the active discussions in these sessions, 47% of participants felt that their clinical practice was changed and 71% felt encouraged to reflect on their learning post-session. All participants who presented submitted workplace-based assessments signed by a named consultant via their electronic portfolios.

Overall, 64% of participants felt that they preferred to engage virtually compared to face-to-face contact and 90% felt that virtual educational meetings played a pivotal role in maintaining team morale and unity during this pandemic period (Figure 7). We reported an overall satisfaction rate of 89% with 87% feeling that they would recommend this bespoke virtual programme to others.

## Discussion

With the rapid change in the hospital workplace, face-to-face contact to deliver educational training to meet the demands of training posed a great challenge during the current pandemic. Moreover, the need to ensure team communication and unity, and to maintain morale through this turbulent time seemed to be of paramount importance. 

Classic face-to-face education has several advantages including easy communication, direct feedback (including non-verbal cues) and re-assuring nature of human touch and encouragement [10]. However, these sessions can be challenging to organise, with regular booking needed to be made several months in advance. Additionally, finding a suitable space to facilitate a large cohort of participants whilst accounting for environmental factors of warmth and seating space may often prove to be a big challenge for any keen department that wants to progress the learning of their juniors. Previous studies have demonstrated various advantages of distance learning compared to the classic face-to-face method of teaching showing comparable outcomes of the two methods for the learner, but better convenience and learner readiness when e-learning was used [11]. 

Our virtual educational tool demonstrates good engagement with regular attendance and active participation and we have found an overall positive experience with engaging with the platform. It has also upskilled many of the participants (particular those in the junior cohort) leading to a more confident approach to non-technical skills such as research & methodology critique and reflective clinical practice, which can often be neglected in traditional teaching sessions.

All sessions provided a structured framework for the active participant to have a goal-oriented approach to their own self-directed learning and allowed many participants to attend in environments where they learned best. Becker C, et al. in their systematic review of outdoor educational systems demonstrate some improvement in concentration, engagement and retention when compared to a traditional in-class model. It was pleasantly surprising to see majority of our participants attending sessions away from the hospital setting [12]. This provided an added layer of convenience and better facilitated those individuals who attended on their rest day periods (voluntarily), thereby avoiding missing educational opportunities. 

We have subsequently been able to recommend this programme to the Trust and have been commended for engaging with educational activities during the pandemic. Moreover, the use of virtual platforms further supported a multidisciplinary approach to learning with several members of the allied specialties such as nurse practitioners, occupational hand therapist and physiotherapists, attending and offering expert advice on key areas pertaining to the topic at hand. This has improved our ability in encouraging interprofessional engagement and learning.

Our results demonstrate a high satisfaction rate with the majority of the members in the department preferring to continue with virtual education over more conventional means whilst we continue to work under the constraints of the pandemic. We accept that not all the requirements of the curriculum can be met virtually with the main drawback being technical skills training and hands-on experience. We also need to consider that virtual presentations take away part of the psychological stress of the individual presenting. That could be attributed to the lack of the physical presentation of the audience and due to the fact that the individual may be presenting from their own 'comfort zone'. Our programme fully accepts this and we have not used this platform for those skills support. 

Our study had two main limitations. The first is the lack of control from before the COVID period as there have been no such studies in our department prior to this pandemic and we have no data to compare with our current findings. The second is the subjective nature of some of our outcomes. More specifically, our finding that some of the participants improved their non-technical skills (presentation, communication and research) is based on their own opinion as documented in the survey. Our follow-up study will be addressing these issues by involving both modules of teaching, since face-to-face teaching sessions are starting again. Our plan is to have both face-to-face and virtual teaching sessions so that we can compare data from both types of teaching at the same time period. We are also going to include more objective assessment tools of presentation, communication and research skills of our participants. 

## Conclusions

In summary, our experience during the pandemic with a pilot virtual education programme has been very positive, maintained an important aspect of training and has had an added effect in maintaining a positive team spirit and comradery during this time.

Virtual education tools are ideal in areas where training spaces may be limited, for example in district general hospitals and to facilitate multi-centric training such as regional and national programmes.

We have demonstrated improvement in non-technical skills and engagement amongst participants in our department and with its flexibility are looking at other opportunities where it may be implemented in the future.
